# A clinical pilot study to evaluate the efficacy of oral intake of phellinus linteus (sanghuang) extract on knee joint and articular cartilage: Study protocol clinical trial (SPIRIT Compliant)

**DOI:** 10.1097/MD.0000000000018912

**Published:** 2020-02-21

**Authors:** Yong Ho Ku, Hyun Lee, Hwa Yeon Ryu, Hae-Jin Park, Mi-Rae Shin, Jae Hui Kang

**Affiliations:** a Department of Acupuncture and Moxibustion Medicine, College of Korean Medicine, Daejeon University; b Department of Herbology; c DHU Bio Convergence Testing Center, Daegu Hanny University Industry Academic Cooperation Foundation.

**Keywords:** knee osteoarthritis, phellinus linteus, randomized controlled trial

## Abstract

**Background:**

Knee osteoarthritis (KOA) is the most common form of degenerative arthritis. We used Phellinus linteus (PL), which has been well-known anti-inflammatory function. In this study, we will evaluate if PL extract improves symptoms with KOA.

**Methods:**

This study will be an 8-week single-center randomized controlled double-blind clinical trial. Total of 24 subjects with KOA will be enrolled and they will be divided into 3 groups, PL 1,000 mg, PL 1,500 mg and placebo. Subjects will be followed up every 4 weeks with efficacy and safety at the 2nd and 3rd visits. All subjects should maintain a dosage schedule for this protocol. The primary outcome will be assessed with the Korean version of the Western Ontario and McMasters Universities. And the secondary outcomes will be measured using the visual analog scale, quality of life scale (EQ-5D-3L), ESR, C-reactive protein, and C-telopeptide of type-II collagen. Statistical analysis will be performed on the principle of full analysis set.

**Discussion:**

This study has inclusion and exclusion criteria and a well-controlled intervention. This clinical trial is the first step to assess the efficacy and safety of PL in patients with KOA. This study will make an important contribution to the literature and aid follow-up research into the use of PL in KOA.

## 1 Introduction

Degenerative arthritis is characterized by pain and limitation of function; it affects the physical mental and social health of the patient^[1]^ and increases socioeconomic costs.^[2]^ In Korea, 43.4% of the elderly have arthritis, and 84.4% encounter problems in their daily lives; therefore, their subjective quality of life is low.^[3]^

Knee osteoarthritis (KOA) is the most common form of degenerative arthritis. Degenerative changes in the articular cartilage occur mostly in people aged >55 years and are exacerbated by mechanical load abnormalities that accompany advancing age, knee joint lesions and injuries, obesity, varus, and valgus deformities, infections, or other arthritis. Pain and discomfort in the knee joint appear initially, and as it progresses, it becomes difficult to walk or stand, and hypertrophy of the synovial membrane, joint effusion, muscle spasms, muscle atrophy, motion restriction, and joint locking may occur.^[4]^

**Figure 1:**
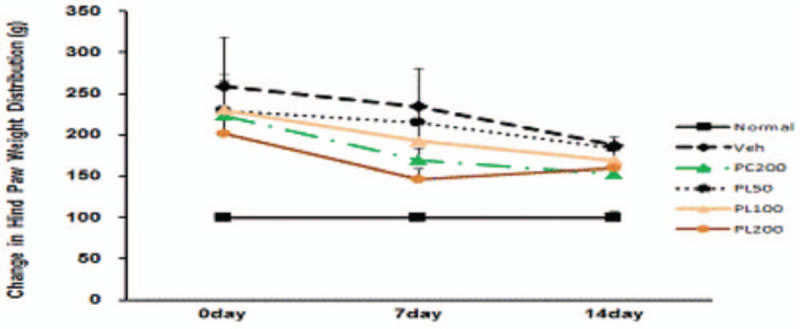
Changes in the relative hind paw weight distribution in rats with monoiodoacetate (MIA)-induced osteoarthritis. All data are expressed means± SEM, n = 6 rats per group. MIA = monoiodoacetate.

Among a variety of beneficial effects, the antiinflammatory function of phellinus linteus (PL) extract is well known, and its specific mechanisms have been actively investigated.^[5]^ In 1 study, PL was been found to reduce the inflammatory response induced by lipopolysaccharide.^[6]^ In another, 200 mg/kg of perna canaliculus extract and doses ranging from 50 mg/kg to 200 mg/kg of PL extract were administered in monoiodoacetate -induced osteoarthritis for 2 weeks. Hind limb weight distribution results showed a significant beneficial effect after 7 days of PL administration, of 100 mg/kg and over. (Table 1, Fig. 1).

**Table 1:**
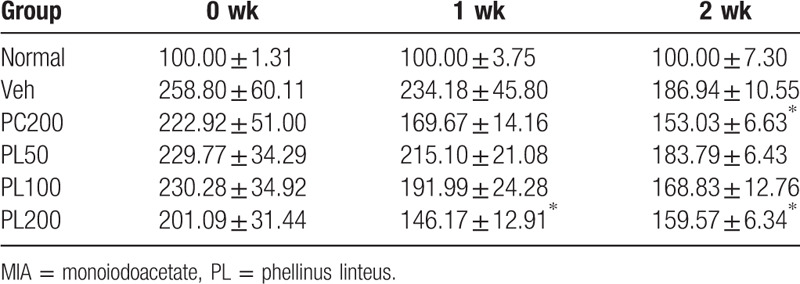
Changes in the relative hind paw weight distribution in rats with monoiodoacetate (MIA)-induced osteoarthritis.

This clinical trial is being conducted based on promising preclinical test outcomes, which suggest PL may be helpful for joint and cartilage health.

## 2 Methods

### 2.1 Study design

This study is a prospective, double-blind, single center, randomized clinical trial to determine the efficacy of PL in KOA. A total of 24 subjects with KOA will be enrolled from the Daejeon University Cheonan Korean Medicine Hospital by advertisements posted on outdoor billboards and our web site. Recruitment started in October 2019, and the trial will end in February 2020. All participants will receive a complete written explanation of the study protocol and informed consent will be obtained by the KMD of the trial.

At the screening, a medical history of the preceding 6 months will be taken and drugs consumed within the last 4 weeks will be recorded. The following blood tests will be done: glucose, complete blood count, blood urea nitrogen, creatinine, total cholesterol, aspartate transaminase (AST), alanine transaminase (ALT), alkaline phosphatase, total bilirubin, gamma (γ)-glutamyl transferase, erythrocyte sedimentation rate (ESR), high-sensitivity C-reactive protein, uric acid, rheumatoid arthritis factor, C-telopeptide of type-II collagen, triglyceride, high density lipoprotein, low density lipoprotein, electrolytes (Na, K, Cl).

Of those who meet the enrolment criteria, 24 healthy patients will be randomly assigned in a 1:1:1 ratio to treatment group 1 (PL 1,000 mg), treatment group 2 (PL 1,500 mg), and the control group (placebo). The interaction will start within 2 weeks after the screening visit.

Subjects will receive PL 1000 mg, 1500 mg or placebo for 8 weeks. The total planned duration of the trial is 8 to 10 weeks. Subjects will be assessed every 4 weeks with efficacy and safety checks at the 2nd and 3rd visits. (Table 2).

**Table 2:**
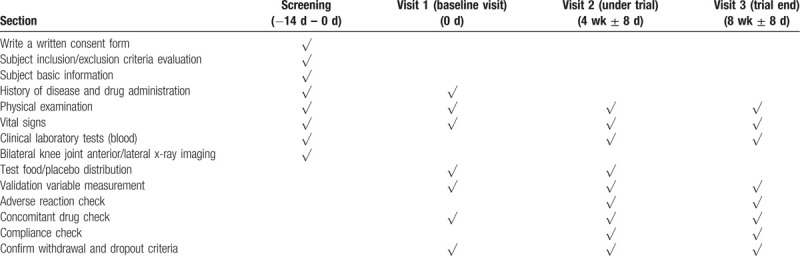
Clinical trial plan.

### 2.2 Inclusion and exclusion criteria

The inclusion criteria are as follows: age, 40 to 75 years; patients with a Kellgren and Lawrence grading scale of 1 or 2 in both knee joints, (assessed on AP and lateral x-rays); those who have taken arthritis medications and have not changed their medications within the last month; applicants who have provided written consent to be a subject of this study because of the effects of KOA on their normal physical activity.

The exclusion criteria are as follows: Those who are currently being treated for clinically significant acute or chronic disorders of any of the following systems: cardiovascular, cerebrovascular, immune, respiratory, hepatobiliary, kidney and urinary, and neurological. Musculoskeletal disorders, mental, infectious or blood and neoplastic diseases. After consideration of the subject's condition, the research director may decide to allow participation in the study. A person whose arthritis is considered to be other than degenerative, as judged by the research director; a history of lower extremity fracture within the last 3 months; creatinine blood levels of more than twice the normal upper limit; patients who have received an intraarticular injection with hyaluronic acid or steroids within 3 months of the study; those whose blood levels of AST GOT or ALT GPT are more than 3 times the normal upper limit; individuals who have uncontrolled hypertension (160/100 mm Hg or more, measurement after 10 minutes at rest); individuals taking medication for a psychiatric disorder, (not including, intermittent medication for sleep disorders); individuals with a history of using herbal medicine or treatment in the last 2 weeks; patients who have received other research drugs within the last 4 weeks; patients who must continue to take drugs that are deemed to affect the results of human clinical trials; persons with a history of gastrointestinal resection (except appendectomy); those who have had artificial joint surgery; pregnant or nursing women; alcoholics or people who drink more than 4 times/week; persons with hypersensitivity to the test food or its components; any person deemed inappropriate by the research director.

### 2.3 Dosage calculation

In the study with SD rats, 100 mg/kg and 200 mg/kg each were administered to rats for 2 weeks and the results showed safety and efficacy in all groups. The effective doses from the animal studies were converted for human administration according to US FDA guidelines for calculating drug effective concentrations based on human body surface area. Effective doses were set at 1000 mg, 2000 mg. Dosages of 100 mg/kg and 200 mg/kg of rats are 960 mg/kg and 1920 mg/kg based on the 60 kg adult intake. This would, therefore, be 2000 mg based on animal test results but since the daily dose of specialty drugs is up to 1650 mg, we have set it to 1500 mg in food. Considering the convenience of preparation and consumption, the intake doses were set at 1000 mg/kg/d and 1500 mg/kg/d.

### 2.4 Sample size calculation

The optimal sample size for the preliminary study and parallel design is 8 people per group. To ensure the feasibility of determining mean and variance with appropriate precision, the total sample size was maintained at 24 and divided into 3 groups.^[7]^ The pilot study will be conducted after the recruitment of 24 subjects (8 per group) who meet the selection criteria.

### 2.5 Randomization and blinding procedures

To conduct this clinical study scientifically and objectively, the final selected subjects are assigned in a ratio of 1:1:1 to experimental groups 1, 2 and a control group using block randomization. The size and number of blocks for randomization are set by the statistician and remain unknown to the researchers. The total random code is generated at about 120% of the target population. The randomized code of 3 digits (eg, PLA-R-001, PLA-R-002, PLA-R-024) is assigned in order of recruitment according to the randomization details. The test compound or placebo, packaged according to the code, is given to the subject. The randomized code cannot be used again even if the target of the randomized code is dropped. Randomization details are generated by statisticians using nQuery Advisor 7.0 (or SAS 9.0 or SPSS 21.0) and sent to the drug manufacturer. The statistician seals the randomized data for each subject in a secure envelope and sends it to the research institution.

To maintain the double-blinding, the placebo and active ingredient are identical and cannot be distinguished by appearance by the participants or staff. The code assigned to subjects will be kept sealed and will not be released until the end of the clinical trial. Cases in which the blinding must be unsealed, such as a serious adverse drug reaction, will be managed using a separate envelope created for each subject so that only their randomization is revealed. Randomization and blinding will not be revealed to the researchers until the end of the study.

### 2.6 Intervention

All participants in the study will receive PL 1000 mg, PL 1500 mg or placebo with water 30 minutes after meals, 3 capsules, twice-daily will be taken for 8 weeks. Subjects will be assessed twice in 8 weeks, at 4 weeks and 8 weeks. The capsules will be produced by Hankookshinyak Pharmaceutical Co. (Nonsan, Republic of Korea).

Any treatment already being taken at the time of the trial or any dietary supplements taken and any additional treatments during the trial will be considered combination therapy. If coadministration is necessary, the details of the administration will be recorded on the case record and subject chart. We will try to avoid any changes to the combination therapy during the trial. Administration of concomitant medications will be minimized during the trial and will be allowed only if it is necessary for the welfare of the subject and it is determined that it will not affect the study according to the judgment of the study personnel. If a surgical operation that does not violate the objectives of this study is performed during the trial, it will be noted on the adverse event page of the case record. The symptoms of overdose in humans are not known, but treatment and other drugs will be provided if adverse reactions or an overdose occurs. Eligible subjects may be dropped if any of the following occurs: violation of inclusion/exclusion criteria, subject requests to withdraw from the study; a serious adverse event or an adverse reaction that makes it difficult to continue; a major violation of the protocol (the use of a prohibited substance, a drug or functional food), or any consumption which may affect the trial or endpoint; subjects become pregnant during the trial. In all cases of dropout, the cause shall be documented in detail in the case record and clinical laboratory tests will also be conducted as a safety assessment. If an adverse reaction occurs, follow-up observation will proceed until the cause of the adverse reaction is identified. The results will be reported in accordance with safety evaluation criteria, evaluation methods and reporting protocols for adverse reactions. The Institutional Review Board will be notified immediately if a subject drops out due to a serious adverse event that is life-threatening, requires hospitalization or prolongation of hospitalization, causes persistent or meaningful disability or dysfunction, congenital malformation, abnormality, medically critical condition, or death.

### 2.7 Outcome measures

The primary outcome will be assessed with the Korean version of the Western Ontario and McMasters Universities (K-WOMAC) osteoarthritis index which is widely used to assess knee pain, joint stiffness, and physical functioning.^[8]^ The secondary outcomes will be measured using the visual analog scale (VAS), Quality of Life Scale (EQ-5D-3L), ESR, CRP, and CTX II. VAS is a widely used tool for pain measurement. Subjects make a mark on a 100 mm straight line (0 mm = no pain, 100 mm = maximum) and the length from “no pain” to the marked point is documented. The test-re-inspection reliability is very high at 0.95.^[9]^ EQ-5D-3L is a health-related quality of life survey questionnaire. ESR is an indicator of the presence of inflammatory diseases or rheumatoid arthritis, CRP is a measure of the progression of an inflammatory disease, and C-telopeptide of type-II collagen is an indicator of bone formation. The primary and secondary outcomes will be evaluated at 4, 8, and 12 weeks after the start of the clinical trial. Safety assessments will be conducted at 4 and 8 weeks. The data of subjects is deidentified and coded by a specific program.

### 2.8 Data collection and monitoring

During the screening visit, the subjects will fill out a questionnaire about their sociodemographic characteristics, provide a medical history of the last 6 months and drug history of the last 4 weeks, and have x-rays of the knee and laboratory tests performed. Personal information and data collected during the screening period will be managed by the hospital. The monitoring of data and research performance will be performed regularly by Jeneolurl Bio Taek Co., Ltd. (Pusan South Korea). The final exam dataset will be accessible to statisticians and key investigators. The results of this study will be published in a peer-reviewed journal.

### 2.9 Statistical analysis

Statistical analysis will be performed on the principle of full analysis set. For the Intention to Treat analysis, missing values will be analyzed by last observation carried forward. The baseline values before treatment and the changes in K-WOMAC at 4 and 8 weeks after treatment will be measured for each of the 2 experimental groups and the control group. To test the difference between two groups of continuous variables, an independent *t* test will be performed if the data follow a normal distribution. If not, Mann-Whitney *U* test will be performed. Paired *t* tests will be performed if the data follow a normal distribution to test the differences between groups. If not, Wilcoxon's signed-rank test will be performed. The test of normality for continuous variables will be analyzed using the Shapiro-Wilk's test. The confidence level will be set at 5%. All statistical analyses will be performed using SPSS Statistics for Windows Version 20.0 (IBM Corp., Armonk, NY).

Drinking, which can affect the weekly evaluation, is considered a management variable, and if there is a difference between groups, analysis of covariance will be performed. Descriptive statistics such as number of subjects, mean, standard deviation, minimum, median, maximum will be presented for baseline, each visit, and final evaluation, as will changes from baseline to final evaluation for continuous data, clinical laboratory tests, and vital signs. For categorical data, a division table will be provided. If necessary, 95% confidence intervals will be calculated. No intermediate analysis will be performed.

Demographic variables measured at the screening visit will be summarized for each treatment group. Assays for comparison of drug administration groups will be performed. The continuous data will be tested using the *t* tests or Wilcoxon's rank sum test. Categorical data will be analyzed using a chi-square test or Fisher exact test.

The safety assessment calculates the rate of adverse events. Continuous data such as comparisons between groups, individual laboratory test results, and biomarkers will be analyzed using paired *t* tests or t-tests with 95% confidence intervals within the group to see if there are any changes compared to the baseline. If the distribution is not normal, nonparametric statistics (Wilcoxon signed rank test or Wilcoxon rank sum test) will be performed. Categorical data are presented as frequencies and ratios for each parameter. The difference between groups will be analyzed using the chi-square test and Fisher exact test.

### 2.10 Withdrawal and dropout

If subjects do not meet the inclusion or exclusion criteria or withdraw their consent, they will be excluded from the study. The researcher will record the reason for their withdrawal.

### 2.11 Safety

The occurrence of side effects will be assessed at each visit. Subjects will be monitored for undesirable, unintended symptoms, signs and diseases. The number and ratio of subjects who have experienced an adverse event will be documented.

### 2.12 Ethics

This study design is based on the Helsinki Declaration and the Korean Clinical Practice Guidelines. This study has been approved by the Korean Institutional Review Board of DUCKMH (number DJUMC-2019-BM11-1) and the study protocol has been registered with the Korean National Clinical Research Information Service (CRIS) (CRIS-KCT0004266). In case of serious adverse events, subjects will be required to withdraw, and this will be reported to the institutional review board of the hospital. Any participant in this study may withdraw consent or voluntarily cease to participate at any time for any reason.

## 3 Discussion

Degenerative arthritis is characterized by loss of articular cartilage, hypertrophy of bone, and production of osteoblasts. It is the most common type of joint disease. Starting at about age 40 years, pathological changes can occur in the articular cartilage but most cases of degenerative arthritis occur at over 55 years and are associated with obesity or family history. Pain and abnormal knee sounds (crepitus) appear early. The onset of pain is gradual and initially comes only during activity, later there is pain at rest. As it progresses, the patient will have difficulty standing, walking, or climbing stairs. Synovial hypertrophy, increased joint fluid, muscle spasms, muscle atrophy, motion limitation, and locked joint are common later symptoms.^[10]^ Treatment of KOA involves medication, physiotherapy, surgery, and injection therapy. Since KOA is a chronic disease, NSAIDs are often used for treatment and they are known to cause gastrointestinal side effects and neurotoxicity.^[11]^ Accordingly, there is a growing interest in herbal medication for KOA.

Among the various benefits of PL, its anti-inflammatory function is well known.^[12]^ This clinical trial is the first step in determining the optimal dosage and duration for PL. Although the sample size of this study is small, we believe it will make an important contribution to the literature and aid follow-up research into the use of PL in KOA. It will provide insights into its clinical therapeutic effect and possible mechanisms of action.

## Author contributions

JHK designed, managed, and oversaw the research protocol and critically revised the manuscript. YHK developed the protocol and wrote the manuscript. Both authors confirmed and approved the final manuscript.

Yong Ho Ku orcid: 0000-0001-7553-4144.
