# A clinical pilot study to evaluate the efficacy of oral intake of phellinus linteus (sanghuang) extract on knee joint and articular cartilage: Study protocol clinical trial (SPIRIT Compliant): Erratum

**DOI:** 10.1097/MD.0000000000021990

**Published:** 2020-08-14

In the article, “Bacteriological pattern and their correlation with complications in culture positive cases of acute bacterial conjunctivitis in a tertiary care hospital of upper Assam: A cross sectional study”,^[1]^ which appears in Volume 99, Issue 8, the authors, Hae-Jin Park and Mi-Rae Shin are being added to the author list.
